# Association between *APOE4* and biomarkers in cerebral adrenoleukodystrophy

**DOI:** 10.1038/s41598-019-44140-3

**Published:** 2019-05-27

**Authors:** Paul J. Orchard, Todd W. Markowski, LeeAnn Higgins, Gerald V. Raymond, David R. Nascene, Weston P. Miller, Elizabeth I. Pierpont, Troy C. Lund

**Affiliations:** 1 University of Minnesota, Division of Pediatric Blood and Marrow Transplantation, 55455 Minneapolis, USA; 2 University of Minnesota, Department of Biochemistry, Molecular Biology and Biophysics, 55455 Minneapolis, USA; 3 Penn State, Pediatric Neurology, 17033 Hershey, USA; 4 University of Minnesota, Department of Diagnostic Radiology, 55455 Minneapolis, USA; 5 University of Minnesota, Division of Clinical Behavioral Neuroscience, 55455 Minneapolis, USA

**Keywords:** Biomarkers, Neurological disorders

## Abstract

Cerebral adrenoleukodystrophy (cALD) is an inflammatory neurodegenerative disease associated with mutation of the *ABCD1* gene. Proteomic analysis of cerebral spinal fluid (CSF) from young males with active cALD revealed markers of inflammation including APOE4. *APOE4* genotype has been associated with an inferior prognosis following acute and chronic neurologic injury. We assessed *APOE4* inheritance among 83 consecutive young males with cALD prior to hematopoietic cell transplant and its association with markers of cerebral disease. The allele frequency of *APOE4* was not significantly different from that of the general population at 17%. Young males with cALD that were *APOE4* carriers had similar CSF protein and chitotriosidase activity to that of non-carriers. In contrast, *APOE4* carriers had an increased burden of cerebral disease involvement as determined by MRI severity score (10.5 vs 7.0 points, p = 0.01), higher gadolinium intensity score (2.0 vs 1.3 points, p = 0.007), inferior neurologic function (neurologic function score 2.4 vs 1.0, p = 0.001), and elevated CSF MMP2 levels compared to that of non-carriers (13168 vs 9472 pg/mL, p = 0.01). These are the first data showing that *APOE4* is associated with increased severity of cerebral disease in cALD and suggest it may be a modifier of disease.

## Introduction

Adrenoleukodystrophy (ALD) is a rare X-linked peroxisomal disease caused by mutations in the very long chain fatty acid (VLCFA) transporter encoded by the *ABCD1* gene, a member of the ATP-binding cassette (ABC) transporter superfamily^1^. Loss of function of *ABCD1* leads to VLCFA accumulation, and adrenal and neurologic dysfunction. Up to 40% of affected young males develop idiopathic, progressive, fatal cerebral inflammation and demyelination (cerebral ALD; cALD) characterized by gadolinium-enhancing T2 lesions on magnetic resonance imaging (MRI); suggesting blood-brain-barrier (BBB) disruption^2^. While hematopoietic cell transplant (HCT) can arrest cALD progression, many young males are diagnosed too late in their disease for transplant to salvage favorable neurologic function^3,4^.

Composed of astrocytes, endothelial cells, and perivascular cells, the BBB is selectively impenetrable unless disrupted through malignancy, injury, or inflammation. Apolipoprotein E (ApoE) is a major lipoprotein found in the plasma via liver secretion and is also found in the CSF secreted by astrocytes^5^. The genetic polymorphisms of *APOE* are *APOE2*, *APOE3* (the most common allele with an allele frequency of 75%), and *APOE4*^6^. Previous work reported by Bell *et al*. has shown that astrocyte-derived ApoE3 protein maintains the BBB integrity through down regulation of the pro-inflammatory protein, cyclophilin A (CypA)^7^. However, when the *APOE4* allele is present, CypA is unregulated (i.e. increased) leading to activation of MMP9 and loss of BBB integrity. This BBB-disruptive mechanism is thought to contribute to the increased risk of Alzheimer’s and Parkinson’s disease in *APOE4* carriers^8–10^.

To explore new biomarkers in the cerebral spinal fluid (CSF), we conducted proteomic analysis of cALD CSF compared to that non-ALD CSF followed by gene ontogeny analysis. In the present study, we identified ApoE family member proteins in the CSF and investigated whether the specific *APOE* genotype (carriers of *APOE4* versus non*-*carriers) correlated with our previously described cALD disease biomarkers.

## Results

### Proteomic analysis of CSF samples

We have previously published several individual CSF biomarkers that are associated with disease burden in cALD^11–14^. In this study, we sought to undertake a comprehensive analysis of the CSF from young males with cALD using a proteomics-based approach. To investigate the proteomic composition of CSF in young males with cALD, we pooled the CSF from 18 young males with cALD prior to bone marrow transplant. A reference sample derived from non-ALD CSF was used for comparison as we have previously published^12,15^. The pooled samples were subjected to trypsin digestion followed by fragment labeling using iTRAQ isobaric tags, separation by liquid chromatography, and peptide identification by tandem mass spectrometry.

**Figure 1:**
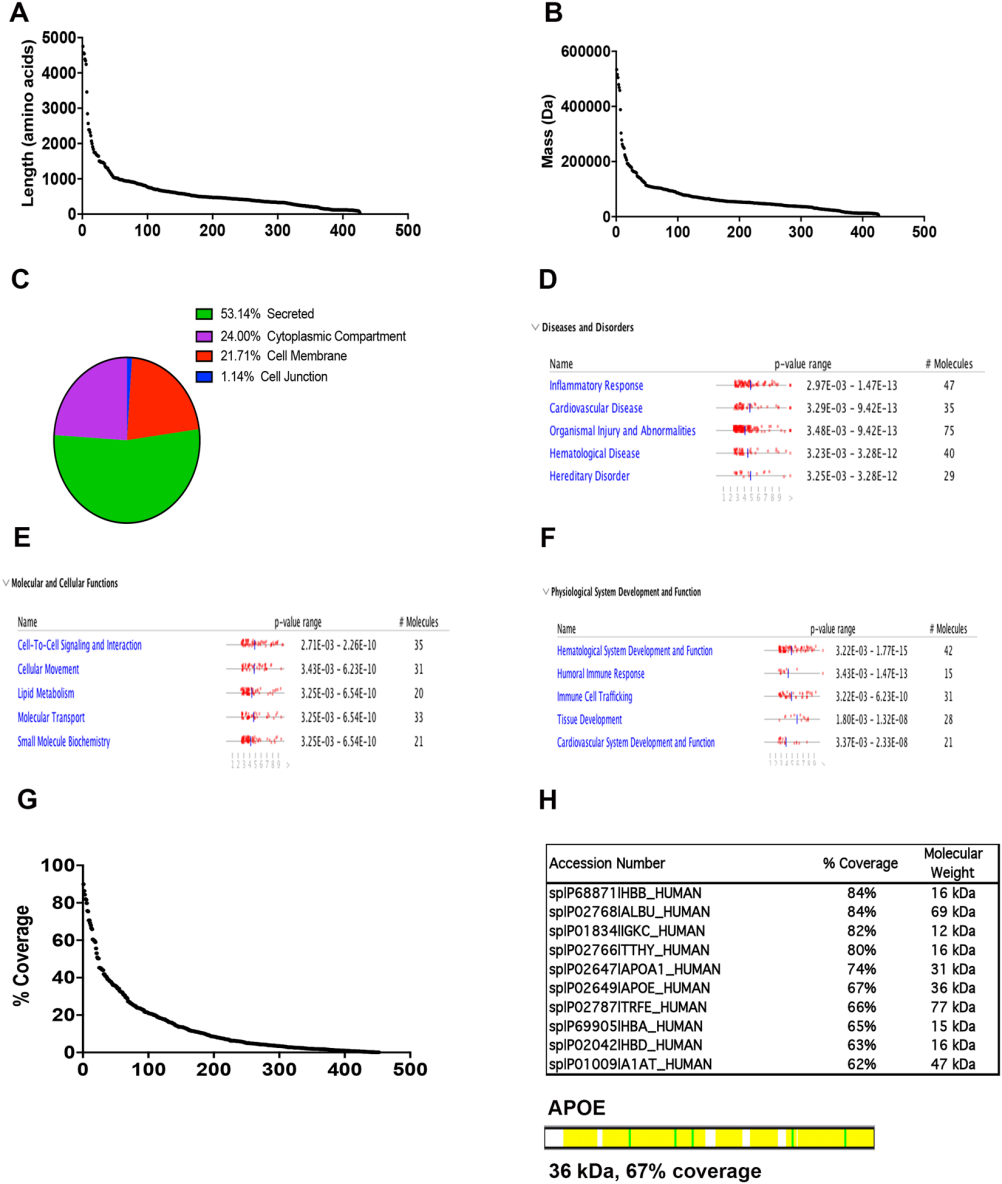
Proteomic evaluation of CSF from young males with cALD compared to non-ALD CSF. (**A**) Amino acid length of identified CSF proteins. (**B**) Mass of identified CSF proteins. (**C**) Uniprot Subcellular location ontogeny terms of CSF proteome. (**D**,**F**) Proteome data were analyzed through the use of IPA (QIAGEN Inc., https://www.qiagenbioinformatics.com/products/ingenuity-pathway-analysis) and shown are the top quartile for Disease and Bio Function analyses: “Diseases and Disorders”, “Molecular and Cellular Functions”, and Physiological System Development and Function” as shown (**B**–**D**). (**G**,**H**) Percent sequence coverage of identified CSF proteins. ApoE protein was determined at a 67% peptide coverage (yellow shading).

With a 1-peptide threshold and 90% confidence setting to identify proteins, we found 450 proteins in the CSF (5316 spectra); the false discovery rate (FDR) was 1%. The median amino acid length for the CSF proteome was 463 (range 45 to 4753) (Fig. 1A). The median molecular weight was 51.8 kDa (range 4.7 to 805 kDa) (Fig. 1B). The number of unique peptides assigned to each protein ranged from 2 to 97 (median of 4). Interrogation of Uniprot subcellular location ontogeny terms indicated that 53.1% of identified proteins were secreted, 24% cytoplasmic, 21% associated with the cell membrane, and 1.1% associated with the cell junction (Fig. 1C).

To understand the ontogeny of the proteins expressed at a higher level in cALD CSF, we performed Ingenuity Pathway Analyses (IPA) from the top quartile of proteins expressed in cALD (Fig. 1D–F). Top quartile proteins in cALD CSF in the Diseases and Disorders category were found to be involved in “Inflammatory Response” and “Cardiovascular Disease” (47 and 35 proteins, respectively). The top Molecular and Cellular Functions were “Cell-to-cell Signaling” and “Cellular Movement” (35 and 31 proteins, respectively). For Physiological System Development and Function, the top ontogenies were the “Hematological System Development and Function” and “Humoral Immune Response” (42 and 15 proteins, respectively).

The protein sequence coverage from MSMS-identified proteins was at an average of 13.7% with a range of 0.2% to 85% (Fig. 1G). We noted that one of the proteins with high sequence coverage from our analysis was ApoE at 67% (Fig. 1H). ApoE exists as 3 isoforms: ApoE2, ApoE3, ApoE4. The single amino acid differences that separate these isoforms were not delineated in the mass spectrum analyses. The most well-studied of these isoforms is ApoE4, a significant risk factor for the development of early Alzheimer’s disease and may play a direct role in blood-brain-barrier (BBB) breakdown^5,7,10,16–18^. Therefore, we sought to determine if ApoE4 played a role in the manifestation of cerebral disease in cALD.

### APOE4 increases endothelial cell oxidative stress and alters mitochondrial function

**Figure 2:**
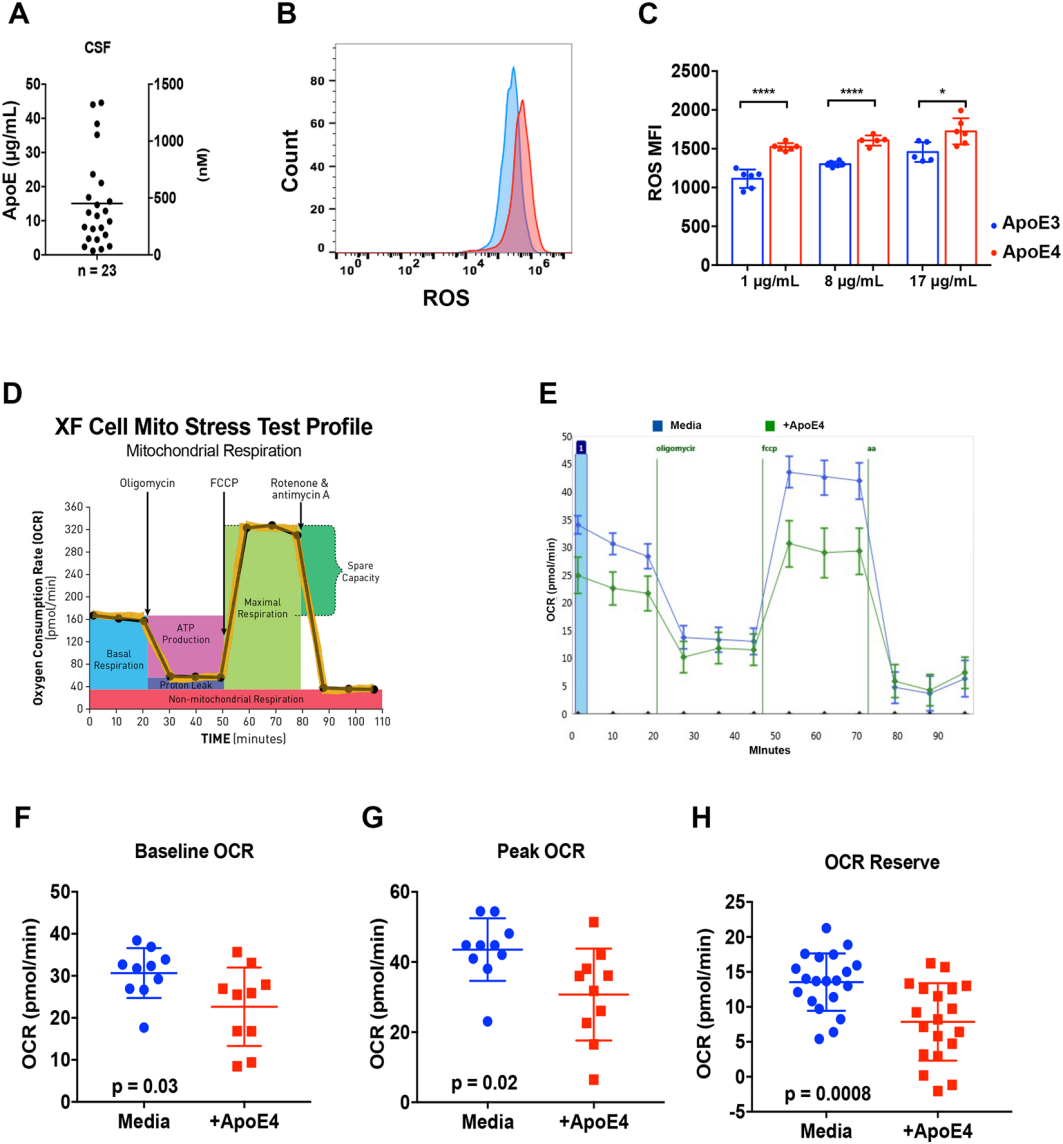
Endothelial energy dysfunction with ApoE4 exposure. (**A**) Total ApoE was determined in the CSF from young males with cALD. (**B**) Representative example of ROS level from HDMECs after 24 hours of exposure to ApoE4 as measured by H_2_DCFDA and flow cytometry (**C**). ROS H_2_DCFDA mean fluorescence intensity (MFI) from HDMECs after 24 hours of exposure to increasing concentrations of ApoE3 and ApoE4. Data was pooled from three independent experiments performed in duplicate. ****p < 0.0001, *p < 0.05 from a Student’s t-est. (**D**) OXPHOS analyses were performed in the XF Extracellular Flux Analyzer (Seahorse Bioscience). Oxygen Consumption Rates (OCR) was measured using XF Assay Kits. The disposable assay kits include a solid-state sensor cartridge embedded with 24 florescent biosensors. The preloaded oligomycin, carbonyl cyanide-4-(trifluoromethoxy)phenylhydrazone (FCCP), and antimycin A were injected sequentially to final concentrations of 0.5 μM for each compound (**E**). Mitochondrial OCR profile from HDMECs after 24 hours of exposure to rApoE4, n = 10–12/group. (**F**–**H**) Quantification of Baseline OCR, Peak OCR, and Reserve mitochondrial OCR.

The presence ApoE4 has been shown to be associated with increased oxidative stress in the brains of affected animals^19^. We evaluated the total ApoE concentration in the CSF of young males with cALD and found a mean of 17 µg/mL, consistent within the range of previously published results^20–22^ (Fig. 2A). ApoE4 has recently been shown to play a role in the maintenance of the endothelial BBB^7^, we next determined if endothelial cells exposed to ApoE4 had elevated levels of ROS. We exposed human endothelial cells (HDMECs) to ApoE4 for 72 hours and measured ROS production using H_2_DCFDA, a dye with fluorescent activity in an increased ROS state (Fig. 2B,C). We found a significant increase in ROS in endothelial cells exposed to ApoE4 compared to ApoE3 with elevated levels of ROS increasing with increasing ApoE concentration (Fig. 2C).

**Figure 3:**
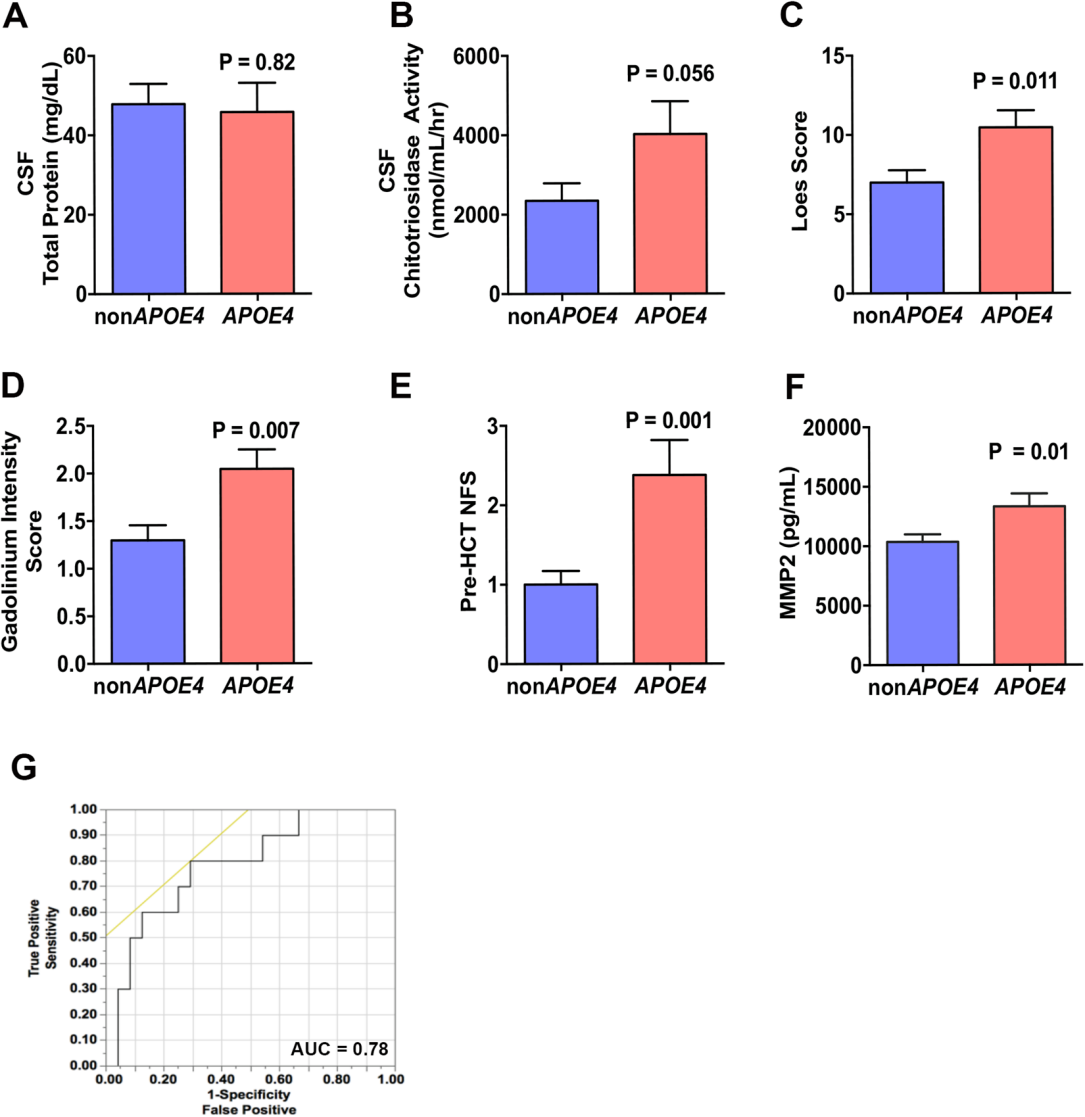
Biomarkers measured in association with *APOE4* inheritance. Biomarkers were assessed during pre-HCT evaluation. (**A**,**B**,**F**) show results from testing of CSF pre-BMT. Scores shown in (**C**,**D**) were obtained from brain MRI within 45 days prior to BMT. Shown are means and standard error of the mean. P-values were generated from a Student’s t-test. (**G**) shows a receiver operator characteristic plot from multivariate analysis of *APOE4* that included the variables: Loes Score, Chitotriosidase, gadolinium intensity, NFS, and MMP2 concentration.

ApoE4 has been previously found to dysregulate energy metabolism and mitochondrial function^23^. We evaluated the effects of ApoE4 on the mitochondrial oxidative phosphorylation (OXPHOS) of human endothelial cells and found that the baseline oxygen consumption rate (OCR) was significantly reduced with the addition of ApoE4 to the cell culture media (30.6 versus 22.7 pmol/min, p = 0.03). Measurement of peak OCR and mitochondrial reserve were also significantly reduced in the presence of ApoE4 (43.5 versus 30.7 pmol/min, p = 0.02 and 13.5 and 7.6 pmol/min, p = 0.008, respectively) (Fig. 3D–H). These data agree with prior reports indicating that ApoE4 can have detrimental effects on endothelial cell physiology and ApoE4 can directly affect ROS and mitochondrial metabolism *in vitro*^23^.

### APOE genotype distribution in cALD

Given that cALD is manifest by blood brain barrier breakdown and cerebral inflammation, we next evaluated young males with cALD who carried the *APOE4* allele to compare the extent of cerebral disease versus non-carriers of *APOE4*. We assessed 83 young males with cALD during pre-treatment evaluations. We found the distribution of *APOE2*, *APOE3*, and *APOE4* alleles to be 0.060, 0.765, 0.175, respectively, which is very similar to that of the general population (Table 1). We observed that only three young males were *APOE4* homozygotes. We performed a Chi-square test for Hardy-Weinberg equilibrium and found p = 0.51 indicating that our allele frequencies were in equilibrium. The mean age of *APOE4* carriers at evaluation was 8.8 years (range 3.8–14.2 years), which was not statistically different from non-*APOE4* carriers at 8.4 years (range 4.5–16.7 years, p = 0.59).

**Table 1:** Distribution of *APOE* alleles in young males with cALD.

Genotype	N = 83	Allele Frequency	
*APOE2/APOE2*	0	*APOE2*	0.060
*APOE2/APOE3*	8	*APOE3*	0.765
*APOE2/APOE4*	2	*APOE4*	0.175
*APOE3/APOE3*	49		
*APOE3/APOE4*	21		
*APOE4/APOE4*	3		

### Correlation of APOE genotype with clinical biomarkers of cALD

Earlier work demonstrated a role for ROS and oxidative stress in ALD^24–26^. Exposure of fibroblasts from patients with ALD to excess levels of fatty acid gives rise to mitochondrial ROS production and reduced mitochondrial respiration as demonstrated by Lopez-Erauskin *et al*.^26^. Given that young males with cALD present with neuro-inflammation and BBB (i.e. endothelial) disruption, we sought to further investigate any modifying effect that inheriting APOE4 could have on patients with cALD. We evaluated CSF total protein concentration, which we previously have described as a biomarker of disease burden^12^, and found that carriers of *APOE4* had a mean CSF total protein concentration of 40.5 mg/dL. This was not different from *APOE4* non-carriers (mean 38.5 mg/dL, p = 0.70) (Fig. 3A). Monocyte/macrophage-produced plasma chitotriosidase has been shown to be biomarker associated with Gaucher disease^27^, and we have previously shown that CSF and plasma chitotriosidase levels are associated with the amount of cerebral disease burden in cALD^14,27^. In the current study, we found CSF chitotriosidase activity in *APOE4* carriers to be 4021 nmol/mL/hr which was higher than that of *APOE4* non-carriers at 2347 nmol/mL/hr, but did not reach statistical significance (p = 0.056) (Fig. 3B).

MRI is the gold standard to determine the extent of cerebral disease in young males with cALD which can be quantified via a 35-point scale based on areas of white matter involvement, aka “Loes Score”^28^. We determined Loes scores of the young males in this cohort and found those carrying *APOE4* had a significantly more MRI white matter involvement with a mean Loes score of 10.5 compared to *APOE4* non-carriers with a mean Loes score of 7 (p = 0.01) suggesting that more demyelination was present in young males carrying *APOE4* (Fig. 3C).

The presence of gadolinium enhancement defines “active” cALD, as it provides evidence of ongoing neuroinflammation. We have recently developed a semi-quantitative method of assessing gadolinium enhancement using a 0–3 gadolinium intensity score (GIS)^11^. A score of 0 indicates no gadolinium enhancement, while scores of 1, 2 or 3 correspond to increasing levels of gadolinium enhancement on MRI. We have shown the GIS to correlates to CSF chitotriosidase, Loes score, and neurologic outcomes after hematopoietic cell transplant (HCT)^11^. In the current study, young males with *APOE4* had a significantly higher mean GIS (mean score 2.0) compared to *APOE4* non-carriers (mean score 1.3, p = 0.007) (Fig. 3D).

Neurologic function is an important indicator of quality of life and ability to participate in everyday activities. We have used a 25-point scoring system developed by Moser and Raymond to assess the neurologic function where points are assessed in several domains when dysfunction is present and a neurologic function score (NFS) is derived^29^ (Table 2). We have previously shown the NFS can be correlated to CSF cytokines, chitotriosidase, Loes score, GIS, and that a higher NFS predicts a more severe outcome after HCT^3,11–14^. As shown in Fig. 3E, *APOE4* carriers had a significantly worse NFS (mean NFS 2.4) as compared to non-carriers (mean NFS 1.0, p = 0.001) indicating more neurologic dysfunction at the time of evaluation.

**Table 2:** The cerebral adrenoleukodystrophy neurologic function score (NFS) used to evaluate gross clinical neurologic status for the cALD cohort pre-transplantation.

Neurologic Function Score	Points
Hearing/auditory processing problems	1
Aphasia/apraxia	1
Loss of communication	3
Vision impairment/fields cut	1
Cortical blindness	2
Swallowing difficulty or other central nervous system dysfunction	2
Tube feeding	2
Running difficulties/hyperreflexia	1
Walking difficulties/spasticity/spastic gait (no assistance)	1
Spastic gait (needs assistance)	2
Wheelchair required	2
No voluntary movement	3
Episodes of incontinency	1
Total incontinency	2
Nonfebrile seizures	1
	25 total

Note that a score of zero denotes absence of clinical signs of cerebral disease. Maximal signs within a domain score the total of all grades within that domain (for example, a patient with “total urinary or fecal incontinency” scores 3, for the sum of “Episodes of incontinency” [1 point] and “Total Incontinency” [2 additional points]).

Prior studies have shown that *APOE4* allows for uninhibited matrix metalloproteinase (MMP) activation in mouse models, which ultimately leads to a permissive BBB^7^. We evaluated the MMP1, 2, 9, 10 concentrations of the CSF and found the *APOE4* carriers had significantly higher levels of MMP2 in their CSF (mean 13168 pg/mL) compared to that of non-carriers (9472 pg/mL, p = 0.01) suggesting that a similar *APOE4* – MMP association may exist (Fig. 3F). There was no significant difference in MMP1, 9, or 10 levels between *APOE4* carrier and non-carriers (not shown). A receiver operating characteristic curve (ROC) from nominal logistic fit analysis indicated reasonable association between the above biomarkers and *APOE4* status (included all biomarkers except CSF Total Protein) (Fig. 3G).

## Discussion

In this large cALD cohort, we report a potential genetic modifier of cALD disease related to the inheritance of the *APOE4* allele. Frequency of *APOE4* inheritance among patients with cALD (17.5%) was slightly higher than the 12% rate reported in the general population^6^, but this difference was not significantly different on Chi-square analysis. Our data indicate that young males with cALD carrying *APOE4* had a higher cerebral disease burden at the time of evaluation for HCT. Our data show a 50% higher average Loes score in the *APOE4* carrier group (i.e., 10.5 vs 7.0). Elevated Loes score in cALD has been shown to be associated with inferior outcomes after HCT^3,30^. Similarly, GIS was also increased in the presence of *APOE4* among the young males with cALD. GIS has also been associated with a higher risk of poor neurologic function after HCT^11^. More intense gadolinium enhancement suggests a more permissive BBB in *APOE4* carriers. Bell *et al*. have shown that a mechanism through which ApoE3 (the most common allele in the general population) may maintain the BBB is via the inhibition of cyclophilin A, an activator of MMP9. It was further demonstrated that carriage of *ApoE4* allowed cyclophilin A to activate MMP9 and led to increased BBB leakiness. Unlike the prior work that showed MMP9 was the key mediator of BBB disruption, we found MMP2 is higher in the CSF of *APOE4* carriers suggesting there may be a difference between murine and human biologic mechanisms. Furthermore, work recently reported by Paik *et al*. demonstrated attenuation of endothelial cell permeability with reduction in Aß induction of MMP-2 expression, suggesting that MMP-2 could be involved in BBB permeability, but it may vary depending on the pathophysiological process (in this case ß-amyloid toxicity). Interestingly, while CSF total protein may be a sign of increased BBB disruption, we did not find an association between *APOE4* and CSF protein. In contrast, we did find an increase in CSF chitotriosidase activity, suggesting that inflammation and BBB disruption is higher in *APOE4* carriers.

It is well described that HCT is the only established treatment modality to stabilize cALD once neuroinflammation has started^3,31–34^. Interestingly, one of the common immunosuppressive drugs given to cALD patients post-HCT to suppress graft versus host disease is cyclosporine, which binds intracellular cyclophilins in T-cells and inhibits their activity^35^. Cyclosporine can also bind and inhibit extracellular cyclophilins such as CypA^36^. Furthermore, Bell *et al*. has shown the *APOE4* transgenic mice treated with cyclosporine had restored BBB integrity^7^. It is interesting to speculate that perhaps one of the mechanisms by which HCT stabilizes cerebral disease in young males with cALD is through the action of cyclosporine on the BBB apart from the allogeneic donor cell engraftment.

Our study was limited in that it was a cross-sectional analysis of young males with a new diagnosis of ALD, and all evaluated patients already manifested cerebral disease. Ideally, future studies should focus on longitudinal evaluation of young males with ALD who do not yet manifest cerebral disease to learn if *APOE4* status truly modulates the onset of cerebral disease as well as learn if there is any change in the velocity of cerebral disease progression observed by serial MRIs and neurologic function.

There are numerous examples in the literature in which *APOE4* is linked to poorer neurologic function following onset of acute or chronic neurologic injury^37–39^. In Alzheimer’s disease, studies have shown a link between *APOE4* inheritance and increased pericyte destruction leading to increased BBB permeability as contributors to the disease process^8^. Persons with neuropsychiatric disease have an increased prevalence of both anti-N-methyl-D-aspartate receptor (NMDA) antibodies and *APOE4*, demonstrating the a between BBB permeability allowing the penetrance of antibody producing immune cells with a neurologic illness^40,41^. Finally, *APOE* genotype can somewhat modify disease onset in metachromatic leukodystrophy, another leukodystrophy often beginning in childhood^42^.

Our data suggest that young males who inherit *APOE4* have more advanced than those who do not carry *APOE4*. We speculate that they may have an accelerated course of disease, but without a true longitudinal study, this is unknown. As newborn screening for ALD becomes more prevalent, longitudinal studies looking at cALD disease modifiers will become increasingly important. Such studies will help inform us which patients may be at greatest risk of developing the cerebral form of the disease, perhaps allowing for increased surveillance with MRI and other biomarker testing.

## Methods

### Participants, ethics approval, and consent to participate

Patients with cALD (n = 83, median age 8.3 years) had CSF sampling done 2 to 6 months prior to hematopoietic stem cell transplant at the University of Minnesota. This study and the use of all bodily fluids were approved by the Committee on the Use of Human Subjects in Research at the University of Minnesota. Informed consent was obtained for all patient samples from the parents or legal guardians on behalf of the child participants. Written assent was also obtained if patients were greater than 8 years of age. the methods were carried out in accordance with the relevant guidelines and regulations.

### Mass spectrometry

#### Protein extraction

CSF samples were concentrated with Amicon Ultra 3 K molecular weight spin filters(Millipore) and buffer exchanged by passing two rounds of 500 µL 0.5 M triethylammonium bicarbonate (Sigma) through the spin columns. The retentates is that a word? from the spin filters was collected and a Bradford assay was done to determine protein concentrations of all samples. Thirty micrograms of each sample was suspended in 160 µl of protein denaturing buffer [7 M urea, 2 M thiourea, 0.4 M triethylammonium bicarbonate (TEAB) pH 8.5, 20% methanol and 4 mM tris (2-carboxyethyl) phosphine (TCEP)] on ice, and the sample was transferred to a PCT tube (Pressure Biosciences, Inc., South Easton, MA) with a 150 µl cap. A Barocycler NEP2320 (Pressure Biosciences, Inc., South Easton, MA) was cycled between 35 kpsi (30 sec) and 0 kpsi (15 sec) for 40 cycles at 37 °C. A volume of 5.83 µl of 200 mM methyl methanethiosulfonate (MMTS) were added to each sample (8 mM final concentration MMTS). The samples were mixed by a brief vortex and incubated (15 min) at room temperature. The sample was transferred to a new 1.5 mL microfuge Protein LoBind tube (Eppendorf, Hauppauge, NY).

#### In-solution digestion

The 20-µg aliquot of each sample was diluted four fold with ultra-pure water. A pooled control sample of 125 µg was made from equivalent amounts of all the samples in the experiment (6.9 µg per sample) and diluted four-fold with ultra-pure water. Trypsin digestion was performed by adding 1:35 ratio of trypsin (Promega, Madison, WI) to total protein. Samples were incubated (16 hrs) at 37 C, frozen at −80 C (30 minutes), and dehydrated *in vacuo*. Each sample was then cleaned with a 4 mL Extract Clean™ C18 SPE cartridge (Grace-Davidson, Deerfield, IL). Eluents were vacuum-dried and suspended in dissolution buffer (0.5 M triethylammonium bicarbonate) from the iTRAQ kit to a final 2 µg/µl concentration.

#### iTRAQ labeling and offline fractionation

20 µg of a research sample or pooled reference was labeled for each iTRAQ channel in the set. A single iTRAQ assay consisted of iTRAQ isobaric tags 113–118 that were used to label CSF proteins from ALD patients 1–6. iTRAQ isobaric tags 119 and 121 were used to label a reference CSF protein extraction from children without adrenoleukodystrophy which we have previously published. The CSF protein from a total of 18 young males with cALD were analyzed in three iTRAQ experiments. The iTRAQ labeling and offline 1^st^ dimension fractionation was done as previously described followed by second dimension separation using liquid chromatography^43^.

#### Liquid chromatography and mass spectrometry

We analyzed the first dimension LC fractions by capillary LC-MS on an Orbitrap Velos MS system as previously described^44^. We made minor modifications to the LC and MS parameters: HCD activation time was 20 msec; lock mass was not employed; dynamic exclusion settings were: repeat count = 1, exclusion list size = 500, exclusion duration = 30 seconds, exclusion mass width (high and low) was 15 ppm and early expiration was disabled.

#### Database interrogation

Tandem mass spectra were extracted by roteome Discoverer version 2.1.0.81. Charge state deconvolution and deisotoping were not performed. All MS/MS samples were analyzed using Sequest (Thermo Fisher Scientific, San Jose, CA, USA; version 2.1.0.81) set up to search the *Homo sapiens* (taxonomy ID 9606) protein FASTA sequences downloaded from UniProt on Dec 13, 2016 after concatenation with the common lab contaminants database from http://www.thegpm.org/crap/ with a total of 92,719 protein sequences. Sequest was searched with a fragment ion mass tolerance of 0.100 Da and a precursor ion tolerance of 50 ppm with trypsin as the enzyme. Methylthio modification of cysteine was specified in Sequest as a fixed modification. Pyro-glutamic acid, deamidation of asparagine, oxidation of methionine, dioxidation of methionine and iTRAQ 8-plex of lysine and peptide N-terminus were specified in Sequest as variable modifications. Quantification results will be reported in a separate manuscript.

#### Criteria for protein Identification

Scaffold (version Scaffold_4.7.3, Proteome Software Inc., Portland, OR) was used to validate MS/MS based peptide and protein identifications. Peptide identifications were accepted if they could be established at greater than 97.0% probability to achieve an FDR less than 1.0% by the Scaffold Local FDR algorithm. Protein identifications were accepted if they could be established at greater than 99.0% probability to achieve an FDR less than 1.0% and contained at least 2 identified peptides. Protein probabilities were assigned by the Protein Prophet algorithm^45,46^.

### *APOE* genotyping

HCT candidates’ *APOE* genotyping was performed on prepared DNA samples by assessing *APOE* single nucleotide polymorphism (SNP) variants rs7412 and rs429358 using the SNP Genotyping Assay (Life Technologies, Grand Island, NY, USA)^44^.

### ApoE concentration

CSF Human Apolipoprotein E was quantified using the enzyme-linked immunosorbent assay: Apolipoprotein E /ApoE Quantikine Kit (#DAPE00, R&D Systems, Minneapolis, MN). The CSF was not diluted.

### Oxidative stress measurement

Human dermal microvascular endothelial cells (HDMECs) were plated in 24-well plate at 10,000 cells/well/mL media +/− 500 nM ApoE4, and incubated for 24 hours at 37C, 5% CO_2_. After incubation, wells were trypsinized with 200 µL of 1x trypsin and washed three times with PBS/0.2% BSA. Cells were next stained with 100 µL CM-H2DCFDA solution according to the manufacturer’s protocol (Thermo Fisher) at room temperature for 5 minutes. Samples were washed three times with PBS/0.2% BSA. 5 µL of 7-AAD (Invitrogen) was added tube for live-dead discrimination. CM-H2DCFDA was read in the FL1 channel; 7AAD was read in the FL3 channel on a BD Accuri C6 analyzer.

### Oxygen consumption rate

HDMECs were plated at 15,000 cells/well +/− 500 nM ApoE4. Plate was incubated at 5% CO_2_, 37 C for 72 hours prior to analysis. The plate was gently washed and each well received 500 µL Assay Medium (non-buffered DMEM without phenol red, 2 mM sodium pyruvate, 25 mM glucose, 1x GlutaMax, pH 7.43) that was pre-warmed to 37 C. The plate was incubated at 37 C for a minimum of 1 hour without CO_2_ as is standard for Seahorse biochemical analysis experiments.

Oxidative phosphorylation determination was performed on a Seahorse XL-24 biochemical analyzer using the following injections: 2 µM oligomycin (PortA), 1 µM FCCP (Port B), 0.5 µM Antimycin A/Rotenone (Port C) and the following parameters: 3 cycles (Mix: 3 minutes, Wait: 2 minutes, Measure: 3 minutes) for each pre-port injection and each post-port injection.

### MMP determination

Samples were analyzed as a multiplex for MMP-1, 2, 9, and 10 using the Luminex platform performed as a multiplex. The magnetic bead set (cat # HMMP2MAG-55K-05, lot 2723323) was purchased from EMD Millipore Corporation (Billerica, MA). Samples were assayed according to manufacturer’s instructions. Fluorescent color-coded beads coated with a specific capture antibody were added to each sample. After incubation, and washing, biotinylated detection antibody was added followed by phycoerythrin-conjugated streptavidin. The beads were read on a Luminex dual-laser fluidics based instrument (Bioplex 200). Samples were run in duplicate and values were interpolated from 5-parameter fitted standard curves.

### Magnetic resonance imaging

Young males with cALD are defined by finding T2-weighted white matter signal abnormalities as well as gadolinium enhancement on MRI. The extent of cerebral involvement (i.e. T2 signal abnormality) can be quantified using a 35-point scoring system develop by Loes *et al*.^28^. Qualitative enhancement was assessed using the gadolinium intensity score (GIS) developed by Miller *et al*.^11^ Loes and GIS scores were determined by a single neuroradiologist (D.R.N.).

### Neurologic function score

Clinical cerebral disease severity was scored at the time of initial patient assessment in clinic by T.C.L., W.M., or G.V.R. using the ALD neurologic function score (NFS)^47^.

### Chitotriosidase activity

Chitotriosidase activity was measured using a modification of the technique described by Sotgui *et al*.^48^. CSF samples were diluted in buffer (10 mM Tris-HCL, 15 mM NaCl, pH 7.5), and 20 μl aliquots of these dilutions were incubated with 20 µl of 22 µM 4-methylumbelliferyl-beta-D-N,N′,N′-triacetyl-chitotriose (MUTAC; Sigma, St. Louis, MO; Cat. #M5639) in 0.5 M citrate-phosphate buffer, pH 5.2, in 0.1% albumin (Sigma, Cat. #A8412) pre-coated 96-well plates for 1 hour at 37 C. The reaction was stopped after 1 hour with 250 µl 0.5 M Na_2_CO_3_-NaHCO_3_, pH 10.7. Enzymatic cleavage of MUTAC produces a fluorescent product, 4-methylumbelliferone (4-MU), which was read on a Molecular Devices, SpectraMAX Gemini fluorometer with 365 nm excitation and 450 nm emission settings. The comparison of relative fluorescent units (RFU) with standards (R&D, Minneapolis, MN; Cat. #3559-GH) ranging from 0.4–12.5 ng/well allowed calculation of activity, which was expressed as nmoles 4-MU generated/mL of CSF per hour^14^.

### Statistical analysis

The functional analyses were generated through the use of IPA (QIAGEN Inc., https://www.qiagenbio-informatics.com/products/ingenuity-pathway-analysis)^49^. Two-way comparisons were analyzed using a Student’s unpaired t-test. Least squares multivariate fit modeling, regression, and nominal logistic fit analyses was performed using JMP (version 13.0). P-values are given in each figure.

## Data Availability

The datasets generated during and/or analyzed during the current study are available from the corresponding author on reasonable request.
